# Mechanism Analysis of Rock Vitrification by Strip Laser Scanning

**DOI:** 10.3390/mi15080973

**Published:** 2024-07-29

**Authors:** Minqiang Kang, Changlang Jiang, Jili Liu, Qihua Zhu

**Affiliations:** 1 Laser Fusion Research Center, China Academy of Engineering Physics, Mianyang 621900, China; jiangcl4814@163.com (C.J.); qihzh@163.com (Q.Z.); 2 Hubei Key Laboratory of Theory and Application of Advanced Materials Mechanics, Wuhan University of Technology, Wuhan 430070, China

**Keywords:** fiber laser, oil and gas exploration, laser drilling, vitrified casing, mechanism, temperature distribution

## Abstract

The process of casing the wellbore in oil and gas drilling consumes a significant amount of time and economic resources. High-energy laser rock fracturing, as an efficient and cost-effective new approach, holds the potential to create a glass-like casing by irradiating the rocks as an alternative to traditional casing. The mechanism behind the vitrification of rocks using laser irradiation, a key factor in achieving glassified casings, remains to be studied. This paper, based on experiments involving scanning sandstone with a line laser, investigates the mechanism of rock vitrification using numerical simulations and X-ray diffractometers. The results demonstrate that the sandstone surface is transformed into glass after laser scanning, with multiple scans and the application of high-speed airflow helping to reduce the formation of bubbles and other phenomena. Furthermore, the speed of laser scanning showed a negative correlation with the laser ablation depth, glass thickness, temperature diffusion rate, and temperature gradient. Based on these findings, a groundbreaking method is proposed for creating high-quality glass by moving the laser to scan the rocks multiple times, offering insights for research into laser-manufactured wellbore casings. Furthermore, this approach holds promising prospects for enhancing and embellishing the exterior of structures and for in situ environmental modifications on planetary surfaces and beyond.

## 1. Introduction

With the escalation of global energy demands, an upsurge in drilling activities is anticipated [1]. Nonetheless, the conventional drilling process is notably time-intensive, with a substantial portion of the time allocated to casing, securing, and ancillary operations [2]. To augment drilling efficiency, the proposition of employing laser drilling techniques has emerged [3]. Noteworthy among its advantages is the glass encasement generated through laser irradiation on rock surfaces, bolstering well wall stabilization, facilitating rock formation separation, and averting wall collapse, thus potentially supplanting traditional casing methods [4]. Furthermore, the corrosion resistance exhibited by the glass casing is commendable. The on-site execution of glass casing using laser technology holds promise in considerably curtailing both drilling durations and expenses.

Recent years have witnessed a surge in scholarly attention towards laser rock fracturing. However, insufficient emphasis persists on rock vitrification using laser techniques. The exploration of the intricate mechanisms underlying laser-induced rock vitrification demands further investigation. Throughout the annals of laser rock fracturing research, temperature has long been deemed a pivotal factor in both rock fracturing and vitrification [2,5,6]. Conversely, laser parameters, rock attributes, and external conditions collectively dictate the resultant state of rock glass formation. Yang et al. [7] discerned distinct stages in rock temperature elevation during laser irradiation, notably delineating rapid, gradual, and equilibrium phases, with prolonged durations observed for gentler temperature increments in rocks rich in refractory constituents, such as quartz. Yan et al. [8] elucidated the facile ionization of metal atoms under laser exposure, culminating in a profusion of non-metallic elements in the glassy compounds. Research on laser-irradiated granite underscores the concentration of temperature rise and molten rock within the fusion zone, culminating in glass formation after a swift cooling process within said domain [9]. After continuous laser exposure, the heat within the fusion zone radiates into the adjacent rocks, triggering a decline in rock integrity and spalling phenomena [10,11]. Chen, Li et al. [12,13] observed the dispersion of numerous cavities and glassy vesicles across the glassy surface derived from molten rock cooling. Regrettably, the compromised rock integrity and spalling instigated by elevated temperatures encumber glass adhesion to rocks, exacerbating the prevalence of air cavities and glass vesicles, thereby compromising glass quality. These challenges impede the application readiness of the resultant glass and underscore the exigency of further investigative endeavors. Existing research primarily focuses on exploring the interaction mechanisms between laser and rocks. However, studies applying laser technology to practical drilling applications remain scarce.

Based on this, our team conducted an investigation into temperature variations when scanning rocks using elliptical and rectangular laser spots. Our findings suggest that compared to circular [13] laser spots, elongated [14] laser spots offer the advantage of larger scanning areas and faster rock removal rates. This preliminary exploration marks a significant advancement in this field. To further explore the mechanisms of laser-induced rock vitrification, we conducted high-speed airflow-assisted rectangular laser scanning experiments on sandstone. This research delved into the relationship between laser movement speed and the formation of vitrified surfaces on rocks. Building upon this, through numerical simulations and phase detection methods, the internal temperature, stress distribution, and vitrification formation process within the rocks were thoroughly investigated. This study ingeniously employed a method of embedding probes within the model to monitor dynamic changes in temperature and stress, speculated based on the characteristics of sandstone according to experimental observations, and validated through numerical simulations. The research outcomes elucidate the mechanisms controlling rock vitrification under laser influence.

## 2. Experimental Part

**Figure 1:**
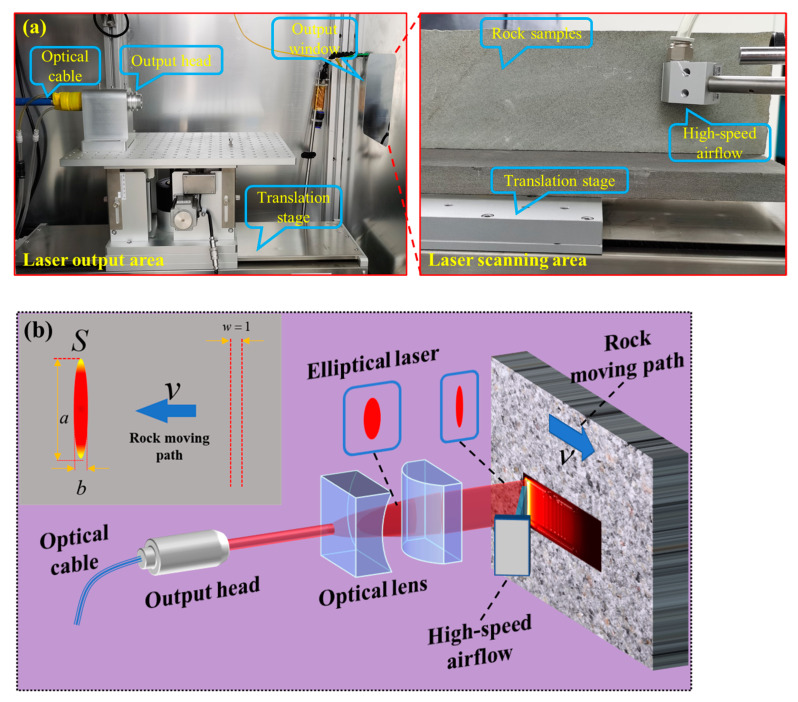
The experimental setup for the laser scanning of rock: (**a**) experimental setup; (**b**) experimental principle.

An illustration of the laser scanning setup is presented in Figure 1a. A circular Gaussian laser beam emanated from a continuous fiber laser (Model: MAX MFSC-1000L, Shenzhen, China), with a peak power of 1 kW and a central wavelength spanning from 1070 to 1090 nm. The laser, configured by the control system, was emitted from the laser head via optical fiber transmission, traversed the optical lenses to generate a strip-shaped laser beam, and was subsequently directed onto the surface of the rock. The manipulation of laser defocus and scanning velocity was orchestrated by governing the three-dimensional displacement platform. The resultant molten rock was eradicated using a high-velocity airflow apparatus (with a maximal gas pressure of 1 MPa), with a vacuum cleaner employed to eliminate rock fragments.

The experimental setup for laser irradiation of the rock is illustrated in Figure 1b. An elliptical laser beam, created by passing a circular laser through an optical lens, was utilized to irradiate a 500 mm × 100 mm × 30 mm sample from Sichuan. After initial experiments, the defocus amount of the laser was set at 120 mm, and the size of the laser spot was 2 mm × 11 mm. Laser scanning speeds of 2.5 mm/s, 5 mm/s, 7.5 mm/s, and 10 mm/s were employed for experimentation. High-speed gas flow (at a pressure of 1 MPa) was used to blow off molten and gasified rocks during the experiment. An X-ray diffractometer (model: Bruker D8 Advance, Ettlingen, Germany) was used to analyze the phase composition of sandstone before and after irradiation.

## 3. Vitrified Morphology

This section encapsulates a description and analysis of the phenomena observed in sandstone experiments under different laser scanning speeds, culminating in a synthesis of partial mechanisms underlying sandstone vitrification. To delve deeper into the mechanisms of sandstone vitrification through laser scanning, a phase analysis of sandstone and its vitrified counterpart was conducted. The relevant analyses and results are explicated below.

### 3.1. Analysis of Surface Morphology

**Figure 2:**
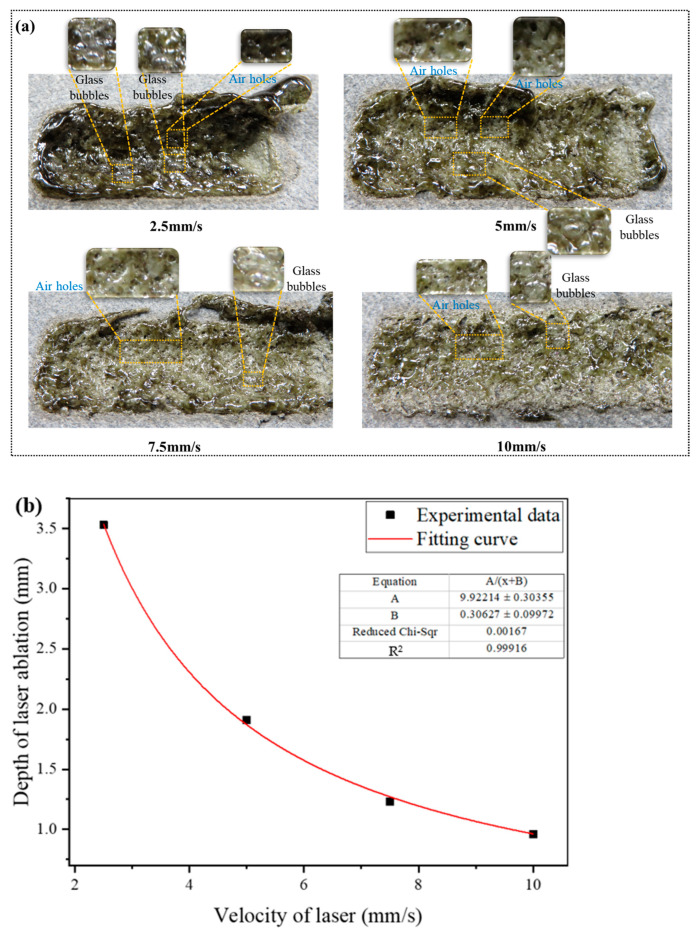
Comparison of sandstone surface morphology at different laser scanning speeds: (**a**) the morphology of sandstone irradiated by laser; (**b**) depth of ablation versus the laser scanning speed.

Figure 2 shows the vitrified surface morphology of sandstone after laser scanning. Under the action of high-speed airflow, the rock melt in the laser irradiation area was blown around the irradiation area, and the remaining rock melt cooled to form a glass layer on the surface of the sandstone. With increasing laser scanning speed, the thickness of the glass covered on the rock surface decreases, the number of air holes on the surface increases, and the number of glass bubble decreases. Glass bubbles are formed when the molten rock is expanded by the gas in the pores of the rock, and the rock is gasified and bulged. As the scanning speed increases, less molten rock is produced, which makes it difficult to form glass bubbles and increases air holes. In the laser scanning experiment, no obvious cracks appeared in the rock, which can be attributed to the porous rock nature of sandstone and the combined effect of high-speed airflow and the scanning laser.

Figure 2b elucidates the nonlinear decrease in ablation depth on the surface of sandstone with increasing laser scanning speed. Due to the latent heat of fusion inherent in rock, a substantial amount of energy absorption is necessitated prior to its melting. Based on the removal principle of rocks under high-energy laser action, at different laser scanning speeds, the laser beam absorbs heat per unit length of the rock (Figure 1b), as shown in Equation (1):

(1) $$Q=\eta \frac{Flux\cdot S\cdot t}{w}=\eta \frac{Flux\cdot S}{v}$$

where Q (J/m) represents the energy absorbed per unit length of the rock, Flux (W/m^2^) represents the laser power density, *t* (s) is the laser irradiation time (=*w*/*v*), S (m^2^) represents the laser spot area, *w* (m) represents the laser beam width, v (m/s) represents the laser scanning speed, and η is the energy absorption rate of rock under laser irradiation.

According to Equation (1), when keeping other conditions unchanged, with increasing laser scanning speed, the absorbed energy of the rock per unit width irradiated by the laser per unit area decreases nonlinearly, which is similar to the variation trend of experimental data in Figure 2b.

### 3.2. Phase Analysis

**Figure 3:**
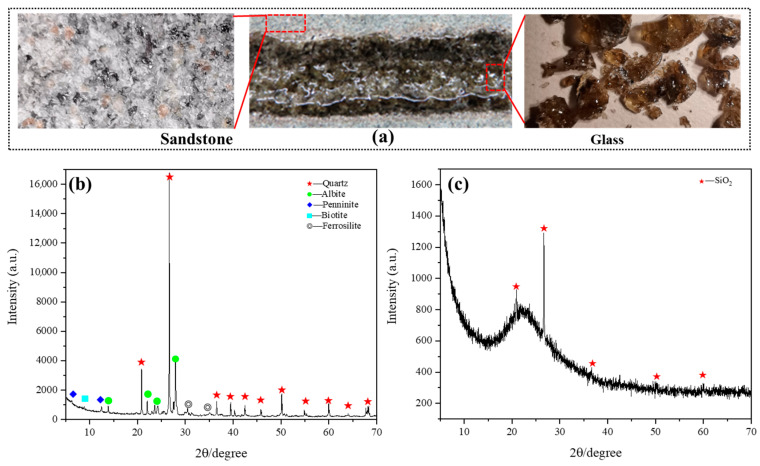
Analysis of sandstone composition after laser scanning: (**a**) sandstone and glass samples; (**b**) XRD analysis of sandstone; (**c**) XRD analysis of glass.

As depicted in Figure 3a, the surface of the sandstone appears predominantly flat, composed of white, black, brown, and transparent mineral particles that are densely distributed. These constituents collectively influence the color, transparency, thickness, and other characteristics of the glass formed when the sandstone is subjected to laser irradiation.

To determine the conversion process of sandstone to glass after laser irradiation, the sandstone and glass were detected using an X-ray diffractometer. The XRD spectrum of the sandstone is shown in Figure 3b. The graph shows that the sandstone is mainly composed of quartz, feldspar, chlorite, iron pyroxene, and biotite, whose main components of the sandstone sample are listed in Table 1. Among them, chlorite and iron pyroxene components cause the rock surface to appear gray–green, and biotite results in uniformly distributed black particles on the sandstone surface. Based on visual examination of the rock glass in Figure 3a, it can be inferred that the rock glass predominantly comprises an amorphous glass matrix with a minor presence of crystalline SiO_2_. From the known results, it can be inferred that mineral components in sandstone, such as quartz (SiO_2_) and feldspar, rapidly melt under high-energy laser irradiation, forming a disordered molten rock melt. After the laser irradiation was stopped, the molten rock in the air environment was rapidly supercooled and did not have enough time to crystallize to form a porous sandstone–glass phase, with only a small amount of SiO_2_ recrystallized. The exclusive presence of the SiO_2_ phase in the XRD analysis of Figure 3c confirms the validity of the aforementioned inference. Among them, the porous morphology of sandstone glass is considered to be caused by partial molten rock gasification and the expansion of air in the original sandstone pores at high temperature.

## 4. Simulation Model Construction

The model comprises two fundamental components: the laser beam model and the sandstone model. Factors influencing the laser beam include spot size, power, and power density distribution. Factors impacting the rock samples include thermal conductivity, specific heat capacity, the melting point, the evaporation point, latent heat of fusion, latent heat of evaporation, elastic properties, Young’s modulus, Poisson’s ratio, and initial constraints. In the subsequent sections, we introduce each constituent of the system and elucidate the modeling process of the transient simulator.

### 4.1. Laser and Rock Model Construction

**Figure 4:**
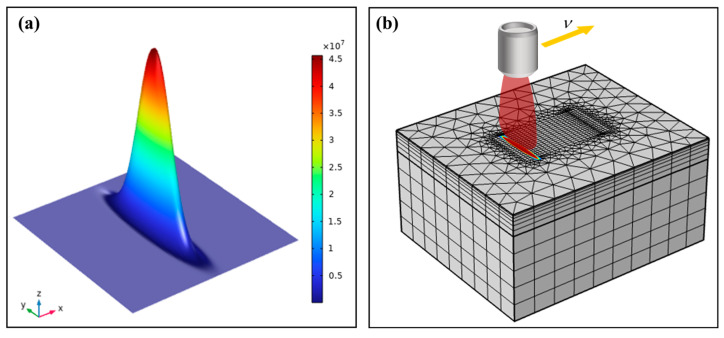
Simulation model: (**a**) elliptical laser beam model; (**b**) sandstone 3D mesh partition model.

The fiber laser beam used in this study was spatially distributed in a Gaussian profile and formed an elongated elliptical spot through an optical lens (Figure 1b). The strip elliptical laser beam model is shown in Figure 4a. The simplified spatial distribution of radial light intensity $I\left(x,y\right)$ is written as follows:

(2) $$I\left(x,y\right)=\eta \frac{iP}{\pi ab}\exp\left(-i\left(\frac{x^{2}}{b^{2}}+\frac{y^{2}}{a^{2}}\right)\right)$$

where $I\left(x,y\right)$ (W/m^2^) represents the light intensity density at any point $\left(x,y\right)$, η is the absorption coefficient of the sandstone to the laser light (indicating the proportion of laser irradiation energy absorbed by the rock), i (1/m) is the light intensity coefficient (detailing the attenuation of optical intensity as the laser beam propagates), *P* (W) is the laser power, a (m) is the laser spot length, and b (m) is the laser spot width.

### 4.2. Determination of Model Material Parameters

The laser ablation of rock is a complex process involving multiphysics changes. The physical parameters of the simplified sandstone model, used to simulate the temperature diffusion and stress field change processes of rock under laser irradiation, are listed in Table 2.

In Figure 4b, the sandstone model was irradiated by a scanning laser beam, and the grid points of the model were used as point heating sources. On each exposed element face, the power density was multiplied by the exposed area, which then spread uniformly to its associated grid points. The equation that produces heat conduction is as follows:

(3) $$k\left(\frac{\partial T^{2}}{\partial x^{2}}+\frac{\partial T^{2}}{\partial y^{2}}+\frac{\partial T^{2}}{\partial z^{2}}\right)+Q_{V}=\rho C_{V}\frac{\partial T}{\partial t}$$

where ρ (kg/m^3^) is the density of the rock sample, $C_{V}$ [J/(kg·°C)] is the specific heat of the rock sample of constant volume, *t* (s) is the time, *T* (°C) is the temperature, $Q_{V}$ (W/m^3^) is the volume heat flux, and *k* [W/(m·°C)] is the thermal conductivity of the rock sample. The boundary conditions used in the numerical simulation in this paper are as follows:

The heat flux of each part of the model is as follows:

(4) $$-k\left(T\right){\left.\frac{\partial T}{\partial n}\right|}_{\Gamma }=h\left(T_{f}-T\right)$$

The thermal radiation at each boundary of the model is as follows:

(5) $$-k\left(T\right){\left.\frac{\partial T}{\partial n}\right|}_{\Gamma }=h_{r}\left(T_{f}^{4}-T^{4}\right)$$

where *T_f_* refers to the initial temperature, *n* is the normal boundary surface, *h* represents the convection heat transfer coefficient, and $h_{r}$ is specified as the radiation heat transfer coefficient.

Thermal stress is caused by thermal expansion due to temperature change, and the thermal stress equation is as follows [16]:

(6) $$\frac{\partial \sigma_{ij}}{\partial t}=2G\left(\frac{\partial \varepsilon_{ij}}{\partial t}-\alpha_{t}\frac{\partial T}{\partial t}\delta_{ij}\right)+\left(K-\frac{2}{3}G\right)\left(\frac{\partial \varepsilon_{ij}}{\partial t}-3\alpha_{t}\frac{\partial T}{\partial t}\right)\sigma_{ij}$$

where $\sigma_{ij}$ (Pa) is the total stress, *G* (Pa) is the shear modulus of the rock sample, $\varepsilon_{ij}$ is the total strain, $\alpha_{t}$ (1/°C) is the linear thermal expansion coefficient, $\delta_{ij}$ is the kronecker delta, and K (Pa) is the bulk modulus of the rock sample.

**Figure 5:**
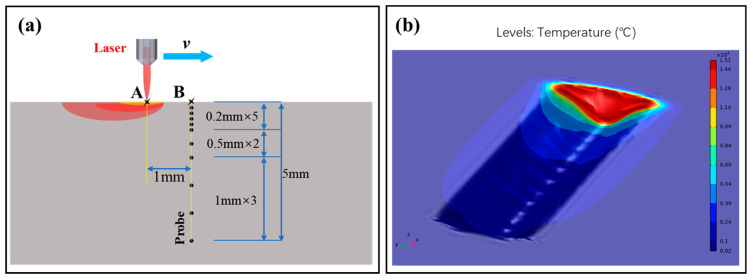
Simulation model configuration and simulation outcomes: (**a**) The distribution of monitor probes in the laser scanning model; (**b**) simulation of the surface morphology of sandstone using laser scanning (*v* = 2.5 mm/s).

Simulating and calculating the dynamics of molten rock under the influence of high-velocity airflow presents a monumental challenge. In order to replicate the authenticity of the experimental setting, streamline the model, and enhance computational efficiency, a supposition is posited, whereby the sandstone swiftly disperses post laser-induced liquefaction. This facilitates the formation of grooves akin to those observed in the physical experiments following sandstone laser treatment. Moreover, to discern the temperature distribution within the sandstone during the laser scanning process, a series of temperature probes was strategically positioned within the sandstone model, as depicted in Figure 5a. As the laser scans the model’s surface at varying speeds, these probes were utilized to measure and capture the internal temperature fluctuations within the rock, offering invaluable insights into the internal thermal dynamics.

## 5. Results and Discussion

This section initially validates the congruence in variation trends between the numerical simulations and experimental data regarding the laser scanning of rocks. Subsequently, it delves into the impact of laser scanning at varying speeds on temperature and stress distribution within the rocks, influencing their vitrification process. Ultimately, key factors governing the vitrification of rocks are summarized.

### 5.1. Laser Ablation Depth Analysis

**Figure 6:**
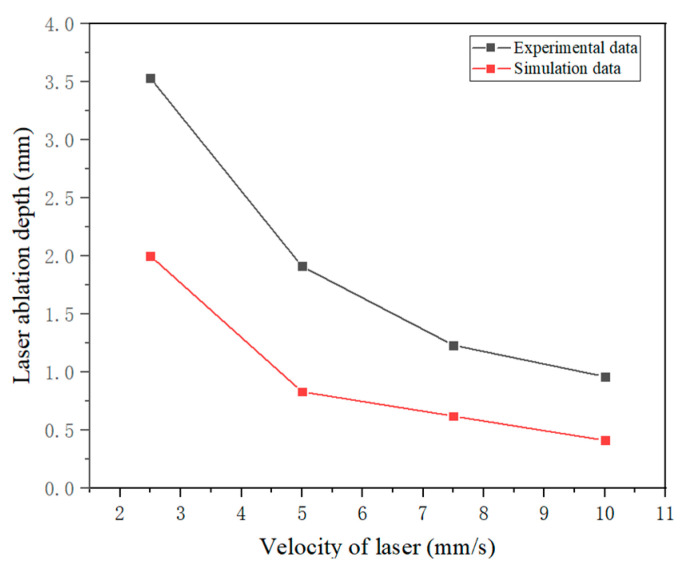
Depth of ablation versus laser scanning speeds.

Upon irradiation, the laser targeted the surface of the sandstone. Once the temperature surpassed the melting threshold of 1450 °C, the molten rock was propelled away with the aid of airflow, initiating the ablation and removal of sandstone to craft grooves, as illustrated in Figure 5b. The fluctuation curve depicting the sandstone ablation depth, as presented in Figure 6, was deduced from the computational simulation of laser-induced ablation on the sandstone. Notably, as the velocity of laser scanning escalated, both the simulated and empirical outcomes exhibited a commensurate nonlinear decline. Figure 6 shows a transformative progression: at laser scanning speeds under 5 mm/s, the ablation depth experienced a rapid diminution correlating with the scanning pace. Conversely, when the laser scan rate surpassed 5 mm/s, the ablation depth diminished at a more gradual pace as the scanning velocity increased. 

### 5.2. Temperature Distribution

**Figure 7:**
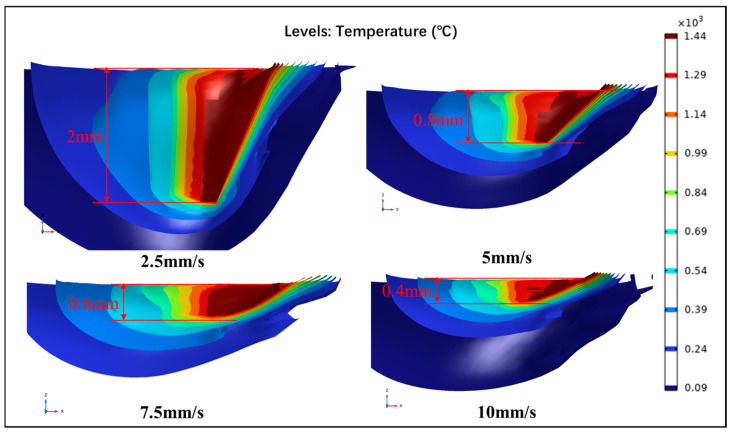
Temperature distribution in sandstone under four different laser scanning velocities.

The temperature profile of laser-irradiated sandstone is illustrated in Figure 7. As the laser scanning speed escalated, the energy absorbed by the laser per unit area of the rock diminished, leading to a reduction in the depth of laser ablation. At a laser scanning speed of 2.5 mm/s, the rock absorbed a substantial amount of energy per unit time, culminating in a swift elevation of rock temperature and the creation of a compact isothermal surface. Conversely, as the laser scanning speed increased, the energy absorption by the rock per unit time decreased, resulting in a sparser formation of the isothermal layer.

**Figure 8:**
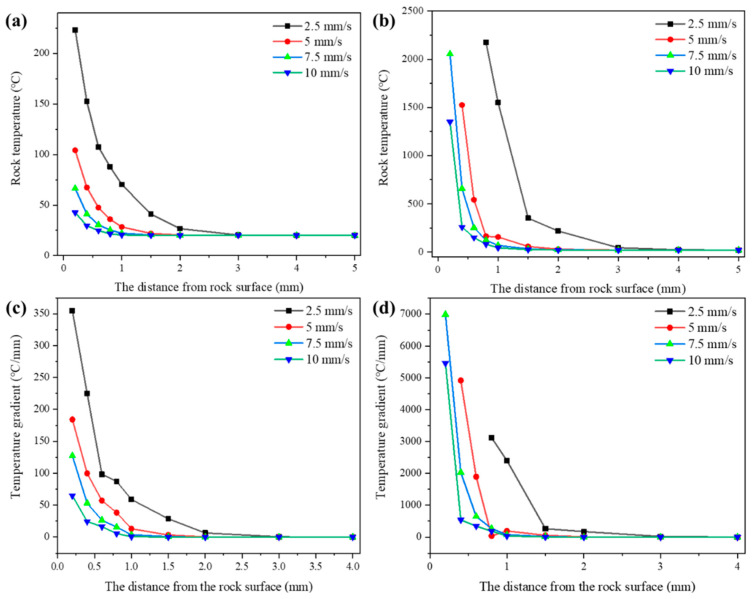
The temperature and temperature gradient from the rock surface during laser scanning: (**a**) the temperature of position A; (**b**) the temperature of position B; (**c**) the temperature gradient of position A; (**d**) the temperature gradient of position B.

As the laser rapidly scaned over positions A and B on the surface of the rock at varying speeds (as depicted in Figure 5a), temperatures of probe points at different distances from the surface were recorded, as illustrated in Figure 8. It is evident upon observation that when the laser scans the surface of the rock, significant temperature variations occur within 3 mm of the rock surface, whereas beyond 3 mm, the temperature impact is minimal.

Figure 8a illustrates that as the laser traversed location A, the rock at the probe point remained unmelted, with the rock temperature exhibiting a sharp ascent as the scanning speed diminished. Upon scanning to position B (Figure 8b), the temperature in the proximity of the rock surface surpassed the rock’s melting point (1450 °C), initiating successive melting of the rock at laser scanning speeds of 2.5 mm/s, 5 mm/s, and 7.5 mm/s.

The temperature gradient of the rock before melting increased as the laser scanning speed decreased (Figure 8c). The analysis based on Figure 8d suggests that the rock’s melting consumes a significant amount of heat energy, resulting in a smaller temperature gradient for the molten rock compared to the unmelted rock. This indicates that as the laser scanning speed increases, the occurrence of rock melting decreases, leading to a larger surface temperature gradient compared to the unmelted rock, making the rock more prone to thermal stress fractures. The temperature gradient at a laser scanning speed of 10 mm/s was smaller than that at 7.5 mm/s, due to the reduced energy absorption per unit area of the rock. Clearly, the variation in laser scanning speed exerts a notable impact on the temperature at a specific point within the rock. By adjusting the laser scanning speed, control over the temperature magnitude and diffusion range on the rock surface can be achieved, influencing the internal stress distribution of the rock.

### 5.3. Stress Distribution

**Figure 9:**
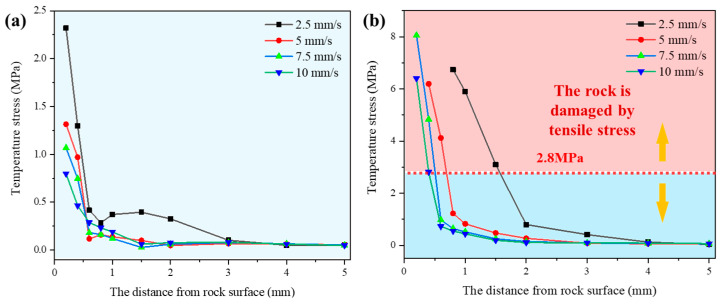
The stress at different points from the rock surface during the laser scan: (**a**) position A; (**b**) position B.

The stress simulations for each probe when the laser scanned through positions A and B are illustrated in Figure 9. At position A, where the rock remains unmelted, the surface stress of the rock decreased as the laser scanning speed increased. It rapidly decreased with depth from the surface, until the decreasing trend slowed down at a distance of 0.6 mm from the surface. As shown in Figure 9b, at position B where the rock has undergone melting, the surface stress of the rock was highest when the laser scanning speed was 7.5 mm/s. The rock’s stress decreased with depth, but the rate of decrease was slower when the laser scanning speed was 2.5 mm/s. The analysis suggests that at scanning speeds of 2.5 mm/s and 5 mm/s, a significant amount of heat energy is consumed by melting of the surface rock, resulting in decreased thermal stress. At a speed of 7.5 mm/s, the rock continuously absorbed heat energy without melting, leading to greater thermal stress. When the scanning speed exceeded 10 mm/s, the rock’s exposure time to laser radiation per unit area decreased, reducing energy absorption and consequently decreasing thermal stress.

The thermal stresses induced in sandstone due to laser irradiation at various speeds consistently remain significantly lower than the failure thresholds of the material (with compressive strength at 46 MPa and tensile strength at 3.5 MPa) [14], aligning well with experimental observations. Sandstone is bound together by abundant quartz particles and possesses numerous internal pores, leading to a higher melting temperature compared to other materials. Moreover, its low thermal conductivity and thermal expansion coefficient contribute to the challenge in generating substantial thermal stresses under laser irradiation, facilitating adherence of the resulting glass phase to the sandstone’s surface.

### 5.4. Glass Shape Control

**Figure 10:**
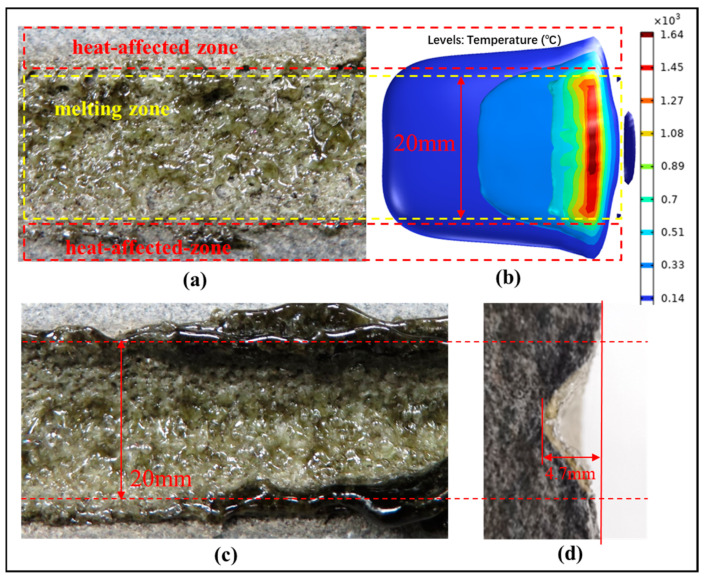
The sandstone’s vitrification through four laser scans is depicted as follows: (**a**) an aerial perspective of a single laser scan, (**b**) temperature contour surface from the simulation, (**c**) an overhead view of four successive laser scans, and (**d**) a cross-sectional display of four successive laser scans.

Following a single laser scan at a speed of 10 mm/s, the sandstone exhibited a glassy surface, as depicted in Figure 10a. Subsequent to laser irradiation and high-speed air blow-drying, although the sandstone adjacent to the glass surface remained unaffected by thermal stress damage, the melted zone on the sandstone surface was encrusted with a textured layer of glass, featuring numerous air cavities and a smattering of glass bubbles. Figure 10b illustrates that the region directly hit by the laser experienced the highest temperatures, leading to rock liquefaction and the creation of a melting zone. The surrounding sandstone was influenced by thermal diffusion from the melting zone, establishing a heat-affected region. Within this heated zone, as temperatures rise, the rocks undergo a transformation process, experiencing phenomena such as liquefaction and gasification, giving rise to glass bubbles and pores. In contrast, the glassy surface of the sandstone resulting from four laser scans at a velocity of 10 mm/s displayed no apparent pores or glass bubbles (as shown in Figure 10c), with the cross-section revealing a consistent glass layer enveloping the rock (illustrated in Figure 10d).

The analysis posits that with successive laser scans on the sandstone, the gas residing in the rock’s pores undergoes thorough heating, expansion, and expulsion, thereby diminishing pore formation and glass bubble occurrence. Supported by high-velocity airflow, the gases within the pores and those generated through rock gasification were swiftly eliminated, hastening the cooling process of the molten rock and facilitating the formation of a sleek glass surface. A comparative examination of the outcomes underscores that employing multiple laser scans in conjunction with high-speed airflow aids in mitigating the emergence of glass bubbles and air voids. Furthermore, the airflow serves to expedite cooling of the molten rock, thereby regulating the glass morphology that develops on the rock’s surface.

## 6. Conclusions

Laser drilling is a groundbreaking innovation with the potential to revolutionize the production of downhole vitrified casings, leading to significant time and cost efficiencies in oil and gas drilling. The sandstone experiment, employing strip laser scanning and the accompanying numerical simulation, detailed in this paper, proves instrumental in unraveling the mechanisms underlying rock vitrification. These findings offer invaluable perspectives to further advance the technology of laser-manufactured glass casings. This article is summarized as follows:

(1) Under laser irradiation, the internal mineral particles within sandstone rapidly melt to form disordered molten rock. Upon cessation of laser irradiation, the molten rock in the ambient air quickly cools, lacking sufficient time for crystallization, resulting in a porous sandstone glass with only a minor amount of SiO_2_ recrystallization. Swift cooling favors the transition of the molten rock to glass, giving rise to a glassy layer intricately embedded on the surface of the rock after laser irradiation.
(2) As the laser scanning speed rises, the ablation depth and glass thickness of sandstone diminish. This reduction can be attributed to the phenomenon of sandstone gasification and the expansion of gases within the pores at elevated temperatures. Consequently, numerous air voids and glass bubbles are prevalent in the cooled glass, ultimately compromising the quality of the glass surface.
(3) Sandstone temperature stress evolves into three distinct stages as the laser scanning speed escalates. Moreover, the rock’s temperature range and diffusion rate are modifiable by the laser scanning speed, fostering enhanced fusion between the glass and rock substrates.
(4) Through the application of multiple laser scans alongside high-speed airflow, the occurrence of air voids and glass bubbles is mitigated. Simultaneously, surplus molten rock is eliminated, facilitating the establishment of a homogeneous glass layer on the rock surface and the realization of a refined glass surface on the rock.

## Figures and Tables

**Table 1:** The components of the sandstone sample.

Mineral	Quartz	Albite	Penninite	Ferrosilite	Biotite
Mass fraction/%	61	30	4	3	2

**Table 2:** Heat physics parameters and mechanical parameters of sandstone [13,15,16,17].

Parameters	Units	Value
Density	kg/m^3^	2.58
Melting temperature	°C	1450
Line expand coefficient	1/°C	A(T) = 4.256 × 10^−14^T^3^ − 6.3 × 10^−11^T^2^ + 3.47 × 10^−8^T
Heat conductivity	W/(m °C)	K(T) = 5.04 × 10^−9^T^3^ + 10^−5^T^2^ − 0.0075T + 3.75
Specific heat capacity	J/(kg °C)	C(T) = 2.17 × 10^−7^T^3^ + 7.5 × 10^−4^T^2^ + 0.306T + 499
Poisson’s ratio	-	0.25
Young’s modulus	Pa	3 × 10^8^
Latent heat of fusion	J/kg	1.8 × 10^6^
Compressive strength	MPa	46
Tensile strength	MPa	3.5
Absorption coefficient	-	0.8

## Data Availability

The original contributions presented in the study are included in the article, further inquiries can be directed to the corresponding author.
